# Transcriptome analysis of polysaccharide-based microbial flocculant MBFA9 biosynthesis regulated by nitrogen source

**DOI:** 10.1038/s41598-020-59114-z

**Published:** 2020-02-19

**Authors:** Lili Fu, Binhui Jiang, Jianwei Wei, Jinliang Liu, Xiaomin Hu, Li Zhang

**Affiliations:** 1 College of Petroleum & Gas Engineering, Liaoning Shihua University, Fushun, 113006 China; 2 College of Resource & Civil Engineering, Northeastern University, Shenyang, 110819 China; 3 College of Municipal & Environmental Engineering, Shenyang Jianzhu University, Shenyang, 110168 China

**Keywords:** Functional clustering, Bacterial transcription, Water microbiology

## Abstract

Microbial flocculant (MBF), an environmentally friendly water treatment agent, can be widely used in various water treatments. However, its use is limited by low yield and high cost. This problem can be solved by clarifying its biosynthesis mechanism and regulating it. *Paenibacillus shenyangensis* A9, a flocculant-producing bacterium, was used to produce polysaccharide-type MBFA9 by regulating the nitrogen source (nitrogen adequacy/nitrogen deficiency). In this study, RNA-Seq high-throughput sequencing technology and bioinformatic approaches were used to investigate the fermentation and biosynthesis of polysaccharide-type MBFA9 by regulating the nitrogen source (high nitrogen/low nitrogen) in the flocculant-producing bacteria *Paenibacillus shenyangensis* A9. Differentially expressed genes, functional clustering, and functional annotation of key genes were assessed. Then the MBFA9 biosynthesis and metabolic pathway were reconstructed. Our results showed that when cultured under different nitrogen conditions, bacterial strain A9 had a greater ability to synthesize polysaccharide-type MBFA9 under low nitrogen compared to high nitrogen conditions, with the yield of MBFA9 reaching 4.2 g/L at 36 h of cultivation. The quality of transcriptome sequencing data was reliable, with a matching rate of 85.38% and 85.48% when L36/H36 was mapped to the reference genome. The total expressed genes detected were 4719 and 4730, with 265 differentially expressed genes. The differentially expressed genes were classified into 3 categories: molecular function (MF), cell component (CC), and biological process (BP), and can be further divided into 22 subcategories. There were 192 upregulated genes and 73 downregulated genes, with upregulation being predominant under low nitrogen. UDP-Gal, UDP-Glc, UDP-GlcA, and UDP-GlcNAc, which are in the polysaccharide metabolic pathway, could all be used as precursors for MBFA9 biosynthesis, and *murA*, *wecB*, *pgm*, *galU*/*galF*, *fcl*, *gmd*, and *glgC* were the main functional genes capable of affecting the growth of bacteria and the biosynthesis of MBF. Results from this study provide evidence that high-level expression of key genes in MBFA9 biosynthesis, regulation, and control can achieve MBFA9 directional synthesis for large-scale applications.

## Introduction

Microbial flocculant (MBF) is an environmentally friendly waste water treatment method with significant potential for development and application, and can possibility replace chemical flocculants. Because of advantages such as its high effectiveness and zero toxicity, with a wide range of applications and no secondary pollution, MBF can be applied to various areas of waste water treatment^1–3^. However, because the flocculant-producing phenotype in the wild-type strain is unstable, low MBF yield and high production costs have greatly limited the production and application of MBF. Many researchers have extensively studied the selection of flocculant-producing bacterial strains, the optimization of growth conditions, the separation and identification of metabolites, treatment of various wastewaters, and the mechanisms of flocculation^4–7^. However, the functional genes in flocculant production, the MBF biosynthesis and metabolic pathways, and regulatory factors in flocculant-producing bacterial strains still remain unclear. Thus, it is difficult to fundamentally solve the problem of how to increase MBF production.

As technologies in molecular biology have rapidly improved, scientists have begun to pay attention to the characteristics of genes involved in the biosynthesis of microbial extracellular polysaccharides and their synthetic pathways. For example, Comstock *et al*. discovered the *wcf* gene cluster, including *wcafA-L*, *wzy*, and *wzx*, which are involved in extracellular polysaccharide synthesis in *Bacteroides fragilis*^8^. This cluster contains genes involved in nucleotide sugar biosynthesis, genes encoding glycosyltransferases, and genes involved in polysaccharide transport. Vorhölter *et al*. showed that in a *Xanthomonas campestris* strain, the biosynthesis of xanthan gum requires UDP-glucose, UDP-glucuronic acid, and GDP-mannose as nucleotide sugar precursors, and the synthesis of xanthan gum repeat units is under the control of a 12-kb gum gene cluster (*genes BCDEEFGHIJKLM*)^9^. Among them, *gum* genes *DHKMI* encode glycosyltransferases. In *Pseudomonas aeruginosa*, 12 structural genes (*algD*, *alg8*, *alg44*, *algK*, *algE*, *algG*, *algX*, *algL*, *algI*, *algJ*, *algF*, and *algA*) form a gene cluster and are assembled in a single operon. *algD* and *algA* are responsible for synthesizing nucleotide sugar precursors, while *algI*, *algJ*, and *algF* are involved in acetylation, and *algG* is involved in epimerization. The *algC* gene is located separately in the bacterial DNA molecule and encodes a phosphoglucomutase involved in the biosynthesis of rhamnolipids and lipopolysaccharides. Key enzymes in the welan gum biosynthetic pathway are PGM, PMI, UGP, TGP, PGI, UGD, with PGM as the key rate-limiting enzyme in welan gum biosynthesis^10,11^.

Polysaccharide-based MBF contains polysaccharides as the main component; the biosynthesis of polysaccharide-based MBF also requires various genes and enzymes. This includes genes involved in monosaccharide synthesis and transfer, nucleotide sugar synthesis, and genes related to the transfer and regulation of the glycosyl group^12^. These genes usually exist as gene clusters in the bacterial chromosome to save energy and increase the efficiency of gene regulation and gene expression^13,14^. In recent years, studies of genomes and transcriptomes have furthered our understanding of polysaccharide synthetic genes and pathways. Based on published reports available, many non-flocculating extracellular polysaccharide synthetic pathways and functional genes have been analyzed for medical, agricultural, or chemical applications^15–18^. However, there are few studies on the synthesis, metabolism, and gene functions of flocculating polysaccharides for water pollution treatment. Research in this field is still at a preliminary stage, and there have not been any complete studies on MBF biosynthetic pathways. In particular, there have been very few reports on bacterial flocculating genes, MBF biosynthesis, and its regulatory mechanisms. This study describes the use of a new strain with high flocculating activity, *Paenibacillus shenyangensis* A9. Based on earlier studies, RNA-Seq technology and bioinformatic approaches were used to study gene transcription and differences in gene expression during bacterial growth and flocculant-producing processes in this strain under different nitrogen conditions (high nitrogen/low nitrogen). The key differentially expressed genes (DEGs) related to polysaccharide synthesis and their functions were here analyzed. Our work identified the polysaccharide-based MBF biosynthetic pathway and its key regulatory nodes, and it validates a previous genome study’s predictions concerning polysaccharide biosynthesis and metabolic pathways. This study identified the high-level expression of key genes in MBFA9 biosynthesis, which can be used to regulate and improve the production of MBFA9 for large-scale applications.

## Results

### Analysis of bacterial growth and MBFA9 yield under different carbon conditions

**Figure 1:**
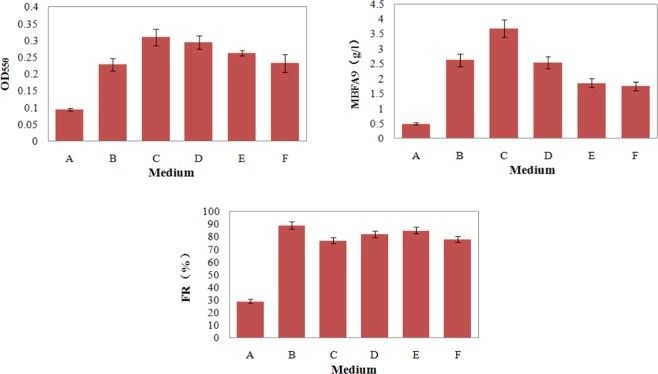
Strain growth, MBFA9 yield and flocculation rate in different carbon source media. (A. General Medium B. Glucose Medium C. Sucrose Medium D. Mannitol Medium E. Lactose Medium F. Mannose Medium).

The bacterial growth, MBFA9 yield, and flocculation rate under selective media containing different carbon sources at 24 h after the activation of liquid bacterial culture are shown in Fig. 1. Glucose, sucrose, mannitol, lactose, and mannose could all be utilized by the flocculant-producing bacteria as the carbon source. The bacterial growth, MBFA9 yield, and flocculation rate were all higher than for those bacteria grown on the general culture medium. In medium containing sucrose, the bacterial growth yield and MBFA9 yield were the highest. At 36 h, the MBFA9 yield was relatively high in medium with glucose, sucrose, and mannitol, and reached 2.62 g/L, 3.68 g/L, and 2.53 g/L, respectively. Except for the general medium, all selective media with different carbon sources had a flocculation rates greater than 77%, while medium containing glucose had the highest flocculation rate of 89%. Thus, although glucose medium did not result in the best bacterial growth and MBFA9 yield, it had the highest flocculation rate. Therefore, using glucose as a carbon source is suitable for bacterial cultivation with the purpose of flocculant production.

### Analysis of bacterial growth and MBFA9 yield under different C/N

**Figure 2:**
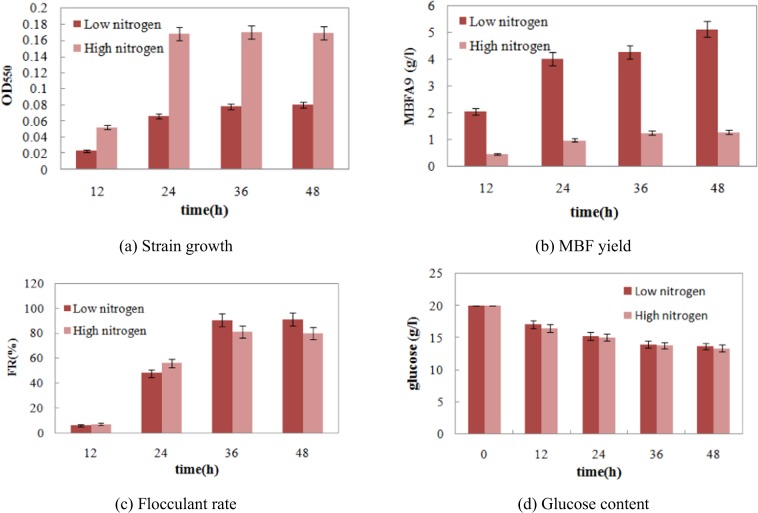
Culture characteristics of nitrogen adequacy/nitrogen deficiency.

Under different nitrogen conditions (different C/N), bacterial growth and MBFA9 yield in bacterial strain A9 were determined (Fig. 2). Under high nitrogen conditions (C/N 10:1), bacterial strain A9 had a higher growth yield but a significantly lower MBFA9 yield than when grown under low nitrogen conditions (C/N 30:1). At 36 h during fermentation under low nitrogen conditions, the MBFA9 yield reached 4.2 g/L.

There were some differences in both bacterial growth and MBFA9 yield under high nitrogen (C/N = 10:1) and low nitrogen (C/N = 30:1) conditions. When carbon and nitrogen nutrition were sufficient, flocculant-producing bacteria A9 reached its optimal growth state. However, this was not the best condition for MBFA9 production. On the contrary, under nutrient deficiency, especially low nitrogen, MBFA9 synthesis and bioflocculability were both higher than those under high nitrogen. The MBFA9 yield and flocculate rate at 36 h reached 4.27 g/L and 90%, respectively. As shown in Fig. 2, while the level of glucose utilized as a carbon source showed that bacterial growth under low nitrogen was not as robust as that under high nitrogen (Fig. 2a), the glucose utilized by bacteria under the two sets of nitrogen conditions was similar (Fig. 2d).

### RNA-Seq quality control and data analysis

After RNA extraction, high quality RNA (RNA total yield > 10 μg, OD_260/280_ = 1.8–2.2) was used for library construction and analysis. The results from RNA samples extracted from flocculant-producing bacterial strain A9 are shown in Table 1. The total RNA samples from H36-L36 passed quality assessment and were qualified for library construction for transcriptome sequencing.

**Table 1:** The spectrophotometric assay of total RNA samples.

Sample	OD260/280	OD260/230	Concentration (ng/µL)	Volume (µL)	Mass (µg)
H36	2.0	2.1	498	25	12.5
L36	1.8	1.6	536	25	13.4

After library construction and sequencing, raw reads from H36-L36 were filtered to generate clean data. Data quality is shown in Fig. S2, including Per Base Quality and Per Sequencing Quality.

Per Base Quality is shown in the left panels, which indicate the log value of the possibility of an erroneous base being called. For example, when the Per Base Quality has a value of 10, it means that the possibility of the wrong base being called is 1/10 (90% possibility being correct). When the Per Base Quality has a value of 20, the possibility of an error is 1/100 (99% possibility being correct), and so on. The X-axis represents the position of bases in the reads, while the Y-axis represents the quality scores of bases. The higher the quality score, the better quality of the base call. Colors in the graph represent different qualities of the base call: very good (green), reasonable quality (orange), and poor quality (red). Examination of the number of bases that fall into different color categories allows assessment of the quality of the base call. Meanwhile, the red line in the graph represents the medium quality score. The yellow box represents quality scores in the 25–75% range. Black whiskers represent the 10–90% range of quality scores. Blue lines represent the mean quality score. The right panels are graphs showing Per Sequence Quality scores, representing the distribution of average quality over the full length of reads. The X-axis represents the average sequence quality score and the Y-axis represents the number of bases corresponding to each quality score. The red line represents the average of quality score per read. In this study, for samples under different C/N conditions and different culturing times, the per base quality for reads between 1–100 bp were all within the very good (green) region, with most of the them lying in the upper area of the very good (green) region. Moreover, most sequencing reads had high-quality scores, therefore high-quality reads consisted of the largest percentage. The quality of the sequencing data in this study was therefore reliable.

### Genome mapping and gene expression analysis

High-quality reads from H36/L36 were mapped to the genome of strain A9. The genome matching results are shown in Table 2. The number of total matched reads from H36/L36 mapped onto the A9 reference genome was 20,820,074 bp and 17,461,770 bp, with a matching rate of 85.48% and 85.38%, respectively.

**Table 2:** Genome matching results in different conditions of H36/L36.

Sample	Clean reads	Mapped on reference	Mapped on gene region	Mapped on intergenic region	Failed to align
H36	24357398	20820074 (85.48%)	7338285 (30.13%)	13481789 (55.35%)	3537324 (14.52%)
L36	20451731	17461770 (85.38%)	8167620 (40.00%)	9294150 (45.38%)	2989961 (14.62%)

**Figure 3:**
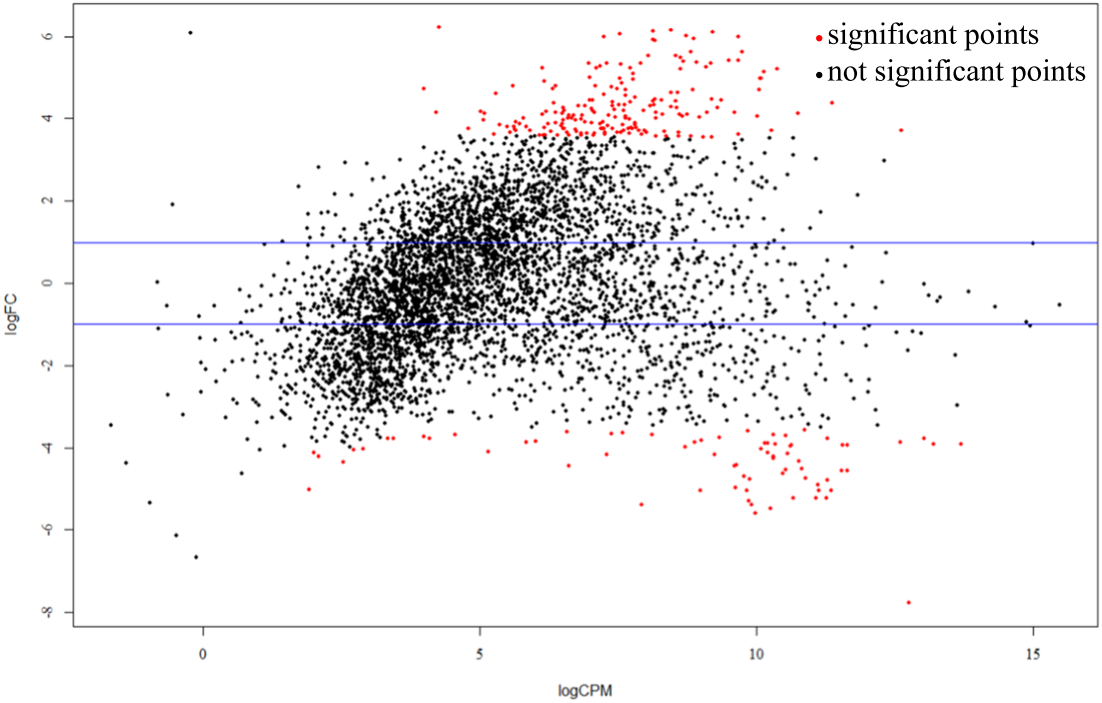
Distribution map of differentially expressed genes.

The mapped reads were then processed using Cufflinks software to calculate the FPKM value of each gene from every sample. The value of gene expression was calculated based on the number of genes in each FPKM value range. P <  = 0.05, |logFC| > 1, FDR < 0.1 was set as the threshold when selecting DEGs. Genes that fulfilled these criteria were considered differentially expressed between two samples. Gene expression levels and DEGs are shown in Table 3 and Fig. 3.

**Table 3:** Differentially expressed genes of L36-H36.

Sample	Total expression	Differential expression	Up-regulation	Down-regulation
L36-H36	4719/4730	265	192	73

Among the 4800 genes predicted by a previous genome study, under different nitrogen conditions at 36 h of bacterial growth, 4719/4730 genes were expressed in the transcriptome data. Compared to the high nitrogen condition, the low nitrogen condition at 36 h had 192 upregulated genes and 73 downregulated genes.

At 36 h during bacterial cultivation, bacterial growth was at the stationary phase, where cellular and extracellular enzymes in the bacteria, as well as the metabolic level in the bacteria, were at the optimal state. Through regulating the C/N ratio, analyzing the functions of DEGs at this phase would be more representative. Thus, the analysis and statistics on DEGs were all based on the data from L36-H36. Sample L36 was the experimental group under low nitrogen (C/N = 30:1), while H36 was the reference group under high nitrogen (C/N = 10:1).

### Functional analysis of DEGs

**Figure 4:**
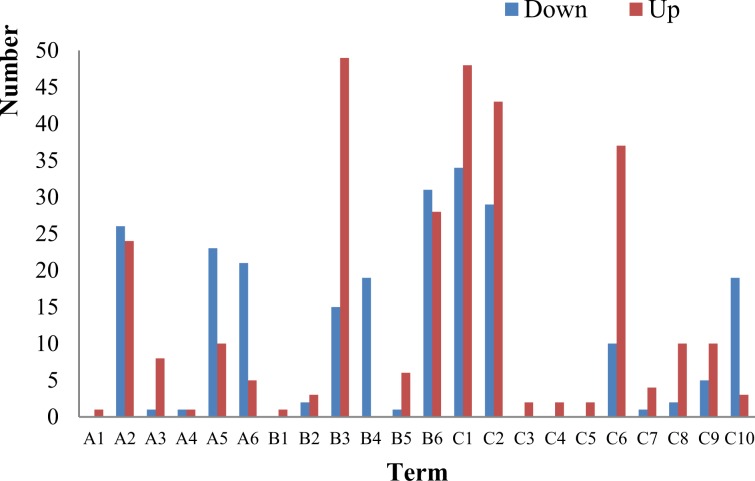
The GO functional enrichment analysis of differentially expressed genes. **CC** A1: extracellular region A2: cell A3: membrane A4: membrane-enclosed lumen A5: macromolecular complex A6: organelle; **MF** B1: protein binding transcription factor activity B2: nucleic acid binding transcription factor activity B3: catalytic activity B4: structural molecule activity B5: transporter activity B6: binding; **BP** C1: metabolic process C2: cellular process C3: signaling C4: developmental process C5: locomotion C6: single-organism process C7: response to stimulus C8:localization C9: biological regulation C10: cellular component organization or biogenesis.

The Gene Ontology (GO) database was used to analyze the functions of gene products in biological processes, cell components, and molecular functions. GO functional enrichment analysis was performed on DEGs between L36 and H36. Results are shown in Fig. 4.

The functions of DEGs were divided among molecular function (MF), cell component (CC), and biological process (BP), with a total of 22 subcategories. At 36 h during the growth of flocculant-producing bacteria under different nitrogen conditions, genes related to CC, MF, and BP all exhibited differential expression to different degrees. First, under low nitrogen, in the CC category, DEGs were concentrated among those related to cell components, macromolecular complexes, and membranes, with the majority being downregulated. This indicated that changes in the level of nitrogen source led to changes in cell components. Second, low nitrogen caused the downregulated expression of all genes related to structural molecules (19/19) in the MF category, and therefore a sharp decrease in the biosynthesis of structural molecules was predicted. In contrast, genes related to transport (6/7) and catalytic activities (49/64) were drastically upregulated. Thus, the activities of enzymes encoded by these genes were increased accordingly. Meanwhile, many genes related to binding activities were differentially expressed, with similar numbers of upregulated and downregulated genes. A difference in expression levels was observed among genes related to binding, suggesting a relatively large change at the molecular level. In addition, in the BP category, genes related to cellular components had significant downregulation, consistent with the trend of downregulation observed in CC. Except for genes related to cellular components that exhibited significant downregulation, other subcategories in BP all exhibited a majority of genes being upregulated, indicating that the corresponding metabolic processes were more active as well.

### Screening of functional genes in the MBFA9 biosynthetic pathway

Through KEGG online annotation, enrichment analysis was performed on the metabolic pathways containing DEGs in the flocculant-producing bacterial strain A9. This was followed by statistical analysis and selection. The predicted 168 metabolic pathways to obtain DEGs related to sugar metabolism, gene expression levels, and the metabolic pathways in which these genes are involved. Here, 42 genes were found to be involved in sugar metabolism, among which 8 genes had significantly different expression levels under low nitrogen, with 7 genes upregulated and 1 downregulated. Information on these genes is shown in Table 4.

**Table 4:** Key genes of carbohydrate metabolism.

Gene ID	Gene	Enzyme	Definition in KEGG	logFC	up/down
Gene28	murA	2.5.1.7	enoylpyruvate transferase	+3.90	up
Gene40	wecB	5.1.3.14	UDP-N-acetylglucosamine 2-epimerase	+4.20	up
Gene22	pgm	5.4.2.2	glucose phosphomutase	+2.33	up
Gene1623	galU/galF	2.7.7.9	UDP-glucose pyrophosphorylase	+4.30	up
Gene1625	fcl	1.1.1.271	GDP-L-fucose synthase	+4.43	up
Gene1626	gmd	4.2.1.47	guanosine diphosphomannose oxidoreductase	+4.47	up
Gene2257 Gene2258	glgC	2.7.7.27	glucose-1-phosphate adenylyltransferase	+4.99 +3.75	up
Gene3296 Gene4611	DPM1	2.4.1.83	dolichol-phosphate mannosyltransferase	−3.74	down

## Discussion

At different nitrogen levels, glucose can serve as a carbon source for both bacterial cell growth and MBFA9 synthesis. Low nitrogen was found to limit bacterial growth to a certain extent, but it increased the ability of the bacteria to synthesize extracellular MBFA9. These results are consistent with those of other studies. For example, it has been reported that extracellular MBFA9 and their polymers, such as curdlan and welan gum, are synthesized in large amounts by bacteria changing their metabolic processes under culture conditions with limited nitrogen^19^. These extracellular MBFA9 are periplasmic, surrounding the bacteria and facilitating adaption to an abnormal environmental conditions. Luedeking^20^ studied the biosynthetic pathway of welan gum and found that a complex set of sources of nitrogen led to more glucose utilized for cell synthesis than a single source of nitrogen. Thus, under a complex nitrogen source, the concentration of the bacterial culture is higher but the yield of welan gum synthesis is lower compared with a single nitrogen source. This indicates that MBFA9 synthesis is related to bacterial growth and that these two processes compete for substrates.

Combined with a previous genomics study by our group on metabolic pathway predictions^21^, our analysis shows that the cause of this difference may be that, under low-nitrogen conditions, flocculant-producing bacteria can regulate the direction of carbon and nitrogen metabolic flow through self-regulatory mechanisms. The limited nitrogen was utilized by cells only under the most favorable and necessary nitrogen metabolic pathways, such as amino acids, to conserve nitrogen^22^. However, it is possible that the carbon source was used not only to support bacterial growth, but also that a large percentage entered the metabolic pathways to supplement amino acid synthesis via self-regulatory metabolism. Eventually, carbon was stored or eliminated as extracellular or intracellular metabolites, i.e., as polysaccharides, to serve as a carbon source and energy storage for the bacteria.

The growth and MBFA9 yield in flocculant-producing bacterial strain A9 exhibited significant differences under different nitrogen conditions. According to the environmental conditions of nitrogen supply, microbes regulate their cellular metabolic systems to achieve the most economical and effective way to survive. The changes in the metabolism inevitably correlate with the differences in gene expression and impact the synthesis of related functional proteins. Thus, through transcriptome studies under different conditions, we could further analyze the differences in metabolism and gene expression levels to more comprehensively determine the mechanism of MBFA9 biosynthesis.

Analysis of DEGs were as shown below.

Enoylpyruvate transferase (2.5.1.7), encoded by the gene *murA*, is a key enzyme in the first step of peptidoglycan biosynthesis. Under high nitrogen, the gene expression of this enzyme was upregulated 3.90-fold. It can catalyze the formation of UDP-N-acetylmuramic acid (UDP-MurNac) from UDP-GlcNAc, while UDP-MurNAc is the precursor for peptidoglycan biosynthesis in the bacterial cell wall^23,24^. This indicated that under low nitrogen, because the increase in UDP-GlcNAc could indirectly lead to increased peptidoglycan synthesis, its precursor UDP-GlcNAc could also be used as a precursor for extracellular MBFA9 biosynthesis.

The *wecB* gene encodes UDP-N-acetylglucosamine 2-epimerase (5.1.3.14). Charles *et al*. in 1985 reported the functions of UDP-ManNac in three bacterial strains^25^. Soldo^26^ found that because teichoic acid is negatively charged, it can bind positive ions from the environment such as Mg^2+^, therefore increasing the concentration of these ions and providing conditions for maintaining the high activity of some biosynthetic enzymes on the cell membrane. Under low nitrogen, the expression of wecB was upregulated 4.2-fold. This could increase the binding ability to Mg^2+^ and further stimulate enzyme activity on the cell membrane, which is favorable for MBFA9 biosynthesis.

Glucosephosphomutase (*pgm*) is a key enzyme in baterial glucose metabolism, as well as MBFA9 synthesis and metabolism. The *pgm* gene encodes glucosephosphomutase (5.4.2.2), which catalyzes the reversible conversion of the phosphate group from C1 to C6 between Glc-6P and Glc-1P. This is the rate-limiting step of glucose metabolism and plays important roles in regulating the reaction direction of Glc-6P in the metabolic pathway^27,28^. Glc-6P can enter glycolysis/gluconeogenesis (P00010) to produce energy (ATP) for bacterial growth as well as reducing power (NADPH). Glc-1P is the donor for UDP-Glc, while UDP-Glc is the glycosyl donor for various metabolic pathways. Thus, glucosephosphomutase is an important enzyme in microbial sugar metabolism. It can be used as an ideal target for constructing genetically engineered bacteria to produce a high level of polysaccharides^29,30^. The *pgm* gene had a 2.33-fold upregulated expression under low nitrogen, suggesting that when nitrogen was sufficient, glucose-6-phosphate was mainly used for energy-producing metabolic pathways such as glycolysis. When nitrogen was low, the expression of pgm significantly increased, causing a high level of conversion of Glc-6P to Glc-1P that led to carbon metabolic flow being distributed to the direction of MBFA9 biosynthesis.

The *galU*/*galF* gene encodes UDP-glucose pyrophosphorylase (2.7.7.9), which catalyzes the interconversion between Glc-1P and UDP-Glc. Its expression was upregulated 4.3-fold under low nitrogen, promoting a high level of UDP-Glc synthesis. UDP-Glc is the precursor for the biosynthesis of different surface structures, lipopolysaccharides (LPS), and microbial extracellular polysaccharides (EPS). It can also be converted to nucleoside diphosphate sugars, pectin, and glycoproteins, which are used to synthesize pectin substances, hemicellulose, glycolipids, and other various glycolysated molecules. The lack of *galU*/*galF* in *Escherichia coli* prevents it from entering the galactose metabolic pathway and causes the loss of normal cell wall and cell membrane formation^31^. Chen *et al*. overexpressed the gene encoding UDP-glucose pyrophosphorylase, which resulted in a 13.3% increase in MBF yield, as well as a 71% increase in flocculation activity^32^. Because UDP-glucose pyrophosphorylase is a key enzyme involved in carbohydrate metabolism and cell wall biosynthesis, this is also a key regualory node for extracellular MBFA9 biosynthesis.

The gene *glgC*, encoding ADP-glucose pyrophosphorylase (EC2.7.7.27), had a 4.99-fold upregulated expression. The enzyme ADP-glucose pyrophosphorylase catalyzes the synthesis of ADP-glucose (ADPG) from glucose-1-phosphate, which serves as a glycosyl donor for glycogen metabolism. Under low nitrogen, glucose-1-phosphate was produced in large amounts; at the same time, the expression level of ADP-glucose pyrophosphorylase was also high. This leads to the biosynthesis of extracellular polysaccharides and the storage of carbon as glycogen, which can be used to synthesize glucose in 5 steps and utilized by re-entering the glucose metabolic pathway.

Fucose synthase (1.1.1.271) encoded by the fcl gene and mannose dehydratase (4.2.1.47) encoded by the *gmd* gene can catalyze the interconversion between GDP-Man and GDP-Fuc. GDP-Man is an important nucleotide sugar in extracellular polysaccharide biosynthesis; it can be converted to other nucleotide sugars as catalyzed by various enzymes. Under low nitrogen, the *fcl* and *gmd* genes had significant upregulated expression. This could further facilitate the conversion to GDP-Man and GDP-Fuc, both of which could be used as precursors for MBFA9 biosynthesis.

The gene *DPM1* encodes GDP-Man:DolP mannosyltransferase (EC2.4.1.83), which catalyzes Dol-P-Man synthesis from Dol-P and GDP-Man. Dol-P-Man is a high-energy mannose residue donor; it can provide mannose residues for polysaccharide biosynthesis^33^. DMP1 exhibited a 3.74-fold downregulated expression, indicating that low nitrogen inhibited the synthesis of Dol-P-Man, therefore resulting in more GDP-Man being converted to GDP-Fuc.

**Figure 5:**
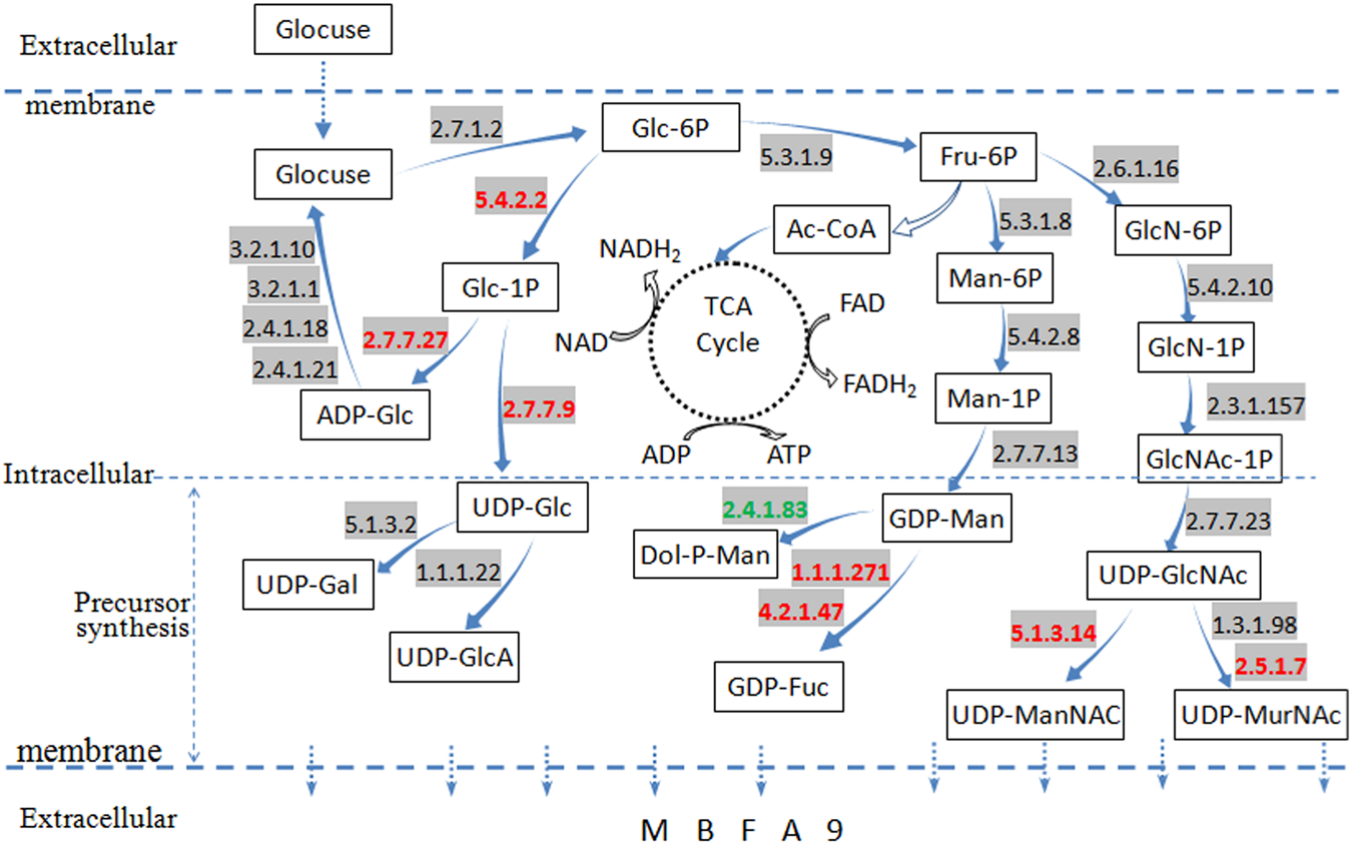
MBFA9 synthesis pathway.

The key DEGs and their key regulatory nodes in metabolism were analyzed. The resulting extracellular MBFA9 biosynthetic pathway is shown in Fig. 5. Red marks indicate upregulated genes. All genes shown in Fig. 5 that were related to MBFA9 biosynthetic enzymes and their characteristics are also shown in Table S1.

Glucose in the medium was phosphorylated to form glucose-6-phosphate (Glc-6P) as catalyzed by hexokinase (2.7.1.2). Glc-6P can be converted through isomerization to glucose-1-phosphate (Glc-1P) as catalyzed by phosphoglucomutase (5.4.2.2). Glucose pyrophosphorylase (2.7.7.9), UDP-glucose dehydrogenase (1.1.1.22), and UDP-glucose epimerase (5.1.3.2) could further catalyze the formation of UDP-glucose (UDP-Glc), UDP-glucuronic acid (UDP-GlcA), and UDP-Gal from Glc-1P. Meanwhile, Glc-1P can also be converted to ADP-Glc as catalyzed by ADP glucose pyrophosphorylase (2.7.7.27), which can be further converted back to Glc and re-enter the cycle as catalyzed by 4 enzymes (ADP-glucose synthase, amylose isomerase, alpha-amylase, alpha-limit dextrinase) in sequence. In the glycolysis/gluconeogenesis (EMP) pathway, Glc-6P can be converted to fructose-6-phosphate (Fru-6P) by glucose-6-phosphate isomerase (5.3.1.9). Fru-6P is an important substance for the synthesis of nucleotide sugars. These are also used for synthesizing extracellular MBFA9. After Fru-6P enters the amino sugar and nucleotide sugar metabolic pathway (P00520), it is first interconverted with glucosamine-6-phosphate (GlcN-6P) as catalyzed by GlcN-6P synthase (2.6.1.16) and glucosamine-6-phosphate deaminase (3.5.99.6). Next, phosphotransferases (5.4.2.10), acyltransferases (2.3.1.157), and UDP-GlcNAc pyrophosphorylase (2.7.7.23) catalyze the formation of UDP-acetylglucosamine (UDP-GlcNAc). UDP-GlcNAc can further be converted to UDP-acetyl-mannosamine (UDP-ManNAc) and UDP-N-acetyl-alpha-D-muramic acid (UDP-MurNAc). Fru-6P can also be converted to UDP-Man by mannose-6-phosphate isomerase (5.3.1.8), phosphomannomutase (5.4.2.8), and GDP-mannose pyrophosphorylase (2.7.7.13), which is then further converted to GDP-Fuc by GDP-D-mannose dehydratase (4.2.1.47) and GDP-fucose synthase (1.1.1.271).

The UDP-Gal, UDP-Glc, UDP-GlcA, UDP-GlcNAc, GDP-Man, and GDP-Fuc in the biosynthetic and metabolic pathway mentioned above can all be used as precursors for MBFA9 biosynthesis. The synthesis of extracellular polysaccharides is then catalyzed by glycosyltransferase^34^. This polysaccharide is the main component of the extracellular polysaccharide produced by the flocculant-producing bacteria A9. Analysis of this polysaccharide metabolic pathway showed that its repeating units include Gal, GlcA, Glc, GlcNAc, Fuc, and Man.

To sum up, the growth of bacteria and MBF biosynthesis were quite different under nitrogen regulation. The ability of polysaccharide synthesis were promoted and the competition of substrate utilization between polysaccharide synthesis and bacteria growth was strengthened when nitrogen deficiency. Flocculent bacteria used self-regulation mechanism to regulate the flow of carbon and nitrogen metabolism. Carbon metabolism related genes changed the transcription level when nitrogen was deficient, and made the carbon metabolism flow into more suitable metabolic pathway to adapt to nitrogen deficiency.

## Materials and Methods

### Strains

Microbial flocculant-producing bacteria *Paenibacillus shenyangensis* strain A9, with high flocculating activity, was isolated from peach tree cultivation soil (10 cm depth) in Liaoning Province, China. The flocculability of fermentation broth of A9 (FBA9) to kaolin solution can reach 96%. It was identified by sequence analysis; its physical and chemical properties and DNA hybridization and fatty acid detection tests indicated that it was a new strain of *Paenibacillus* sp.^35^. The strain was preserved in the Preservation Center of the Institute of Microbiology, Chinese Academy of Sciences (Preservation No. CGMCC2040).

### Culture media

A single colony was inoculated into 100 mL medium A consisting of 5 g beef paste, 10 g peptone, and 5 g NaCl in 1 L distilled deionized water (initial pH 7.2−7.5). After incubation for 24 h at 30 °C, 1 mL fermentation broth of medium A was inoculated into 100 ml carbon source selection medium B and high nitrogen/low nitrogen medium. Medium B consisted of 20 g glucose/sucrose/D-mannose/lactose/mannitol/D-fructose, 1 g yeast extract, 5 g K_2_HPO_4_, 2 g KH_2_PO_4_, and 0.1 g NaCl in 1 L distilled deionized water (initial pH 7.2–7.5). High nitrogen/low nitrogen medium consisted of 20 g glucose, 2 g/0.67 g (C/N 10:1/30:1) yeast extract, 5 g K_2_HPO_4_, 2 g KH_2_PO_4_, and 0.1 g NaCl in 1 L distilled deionized water (initial pH 7.2–7.5). Then, medium B and high nitrogen/low nitrogen medium were incubated for 36 h at 30 °C for detection of fermentation broth indicators.

### Indicators and analysis methods

Strain biomass: 1 mL of fermentation broth with different culture times (0 h, 4 h, 8 h, 12 h, 24 h, 36 h, 48 h, and 60 h) was diluted into 10 mL and mixed evenly. The absorbance value (OD) was measured at 660 nm with distilled water as a blank reference, and growth curves were drawn to show the growth of bacteria.

MBFA9 weight: FBA9 was diluted three times and centrifuged at a speed of 8000 rmin^−1^ for 15 min to remove the bacteria, then concentrated in a rotary evaporator, precipitated by two volumes of anhydrous ethanol, and placed at 4 °C for 24 h. After mixing, filtering, and collecting the precipitate, the above operations were repeated with one volume of anhydrous ethanol. The white flocculants were collected twice and frozen for 24 h in an ultra-low temperature refrigerator, and then lyophilized into dry powder MBFA9 by a freeze dryer.

The flocculation rate (FR): Kaolin (0.5 g) was placed in a 100 mL graduated cylinder, to which 100 mL distilled water was added. This was shaken well, and 0.1 mL FBA9 was added. After repeated shaking, it was rested for 10 min. The supernatant of 50 mL was analyzed by an ultraviolet spectrophotometer at OD_550_. The procedure without FBA9 under the same conditions was used as a blank control. The formula for calculating flocculation activity, expressed as the flocculation rate (FR), was

1 $${\rm{FR}}( \% )=\frac{{\rm{A}}-{\rm{B}}}{{\rm{A}}}\times 100 \% $$

where A is the optical density of the kaolin suspension without FBA9 at 550 nm, and B is the optical density of the kaolin suspension with FBA9 at 550 nm.

### Transcriptome sequencing by RNA-Seq and data analysis

RNA-Seq transcriptome sequencing and bioinformatics analysis of sequencing data were carried out on strains H36/L36 cultured for 36 h under different nitrogen conditions. The workflow is shown in Fig. S1.

#### RNA-Seq

The TRIzol® Reagent (Invitrogen) RNA extraction kit was used to extract total bacterial RNA from the fermentation broth. RNA samples were then treated with DNase I (TaKara) to eliminate residual genomic DNA. RNA quality was examined using a NanoDrop2000 (NanoDrop Technologies) microvolume UV-Vis spectrophotometer. Samples that passed quality assessment were used for cDNA library construction for RNA-Seq. The cDNA library was constructed using an RNA-Seq sample preparation kit (TruSeq RNA Sample Prep Kit) following the instructions from the manufacturer (Illumina). RNA-Seq was performed on a Hiseq2500 platform using paired-end (PE100), 2×100 bp sequencing. The datasets supporting the conclusions of this article are available in the SRA repository (Samples in BioProject PRJNA540204) in https://www.ncbi.nlm.nih.gov/sra.

#### RNA-Seq data analysis

The preliminary FASTQ data from RNA-Seq was subjected to quality control and the removal of redundant and low-quality reads using SeqPrep (https://github.com/jstjohn/SeqPrep) and Sickle (https://github.com/najoshi/sickle) software. High-quality sequencing results (clean data) were then obtained. The software Bowtie 2.0 (http://bowtie-bio.sourceforge.net/index.shtml) was then used to match the high-quality reads to the reference genome. The DEGseq R package was used to analyze the FPKM values of the genes mentioned above. DEGs in the sample genome were identified using assigned parameters of P <  = 0.05, |logFC| > 1, and FDR < 0.1 as thresholds.

Blast2GO software was used to BLAST DEGs against the Gene Ontology (GO) database in NCBI for GO functional enrichment analysis. The purpose of GO enrichment analysis was to discover the GO functional categories of significantly upregulated or downregulated genes. Differences in the expression of genes from these GO categories are important factors that lead to phenotypic differences in different samples.

For enrichment analysis of metabolic pathways that DEGs were involved in under different culturing conditions, the automatic annotation server of KEGG (KASS, http://www.genome.jp/tools/kaas/) was used.

## Supplementary information

Supplementary Information.

## Data Availability

The datasets generated during and/or analysed during the current study are available from the corresponding author on reasonable request.
