# Supplementary material for: Transcriptome analysis of polysaccharide-based microbial flocculant MBFA9 biosynthesis regulated by nitrogen source
Source: Sci Rep. 2020 Feb 19;10:2918. doi: 10.1038/s41598-020-59114-z (PMC7031244; doi:10.1038/s41598-020-59114-z)
Supplement: Supplementary file 1 — Supplementary Information. [file 41598_2020_59114_MOESM1_ESM.pdf]

SREP-19-29856A

Transcriptome analysis of polysaccharide-based microbial flocculant MBFA9 biosynthesis regulated by nitrogen source

Lili Fu[1,*], Binhui Jiang[2], Jianwei Wei[1], Jinliang Liu[1], Xiaomin Hu[2]，Li Zhang[3]

[1] College of Petroleum & Gas Engineering, Liaoning Shihua University, Fushun, 113006, China

[2] College of Resource & Civil Engineering, Northeastern University, Shenyang, 110819, China

[3] College of Municipal & Environmental Engineering，Shenyang Jianzhu University，Shenyang，110168，China

[*] fulili@lnpu.edu.cn

Legends to the supplementary figures/tables

Fig. S1 Flow of RNA-Seq transcriptome sequencing and informatics analysis

Fig. S2 Sequencing data quality control results under different nitrogen conditions

Table S1 Key enzymes in MBFA9 synthesis and properties

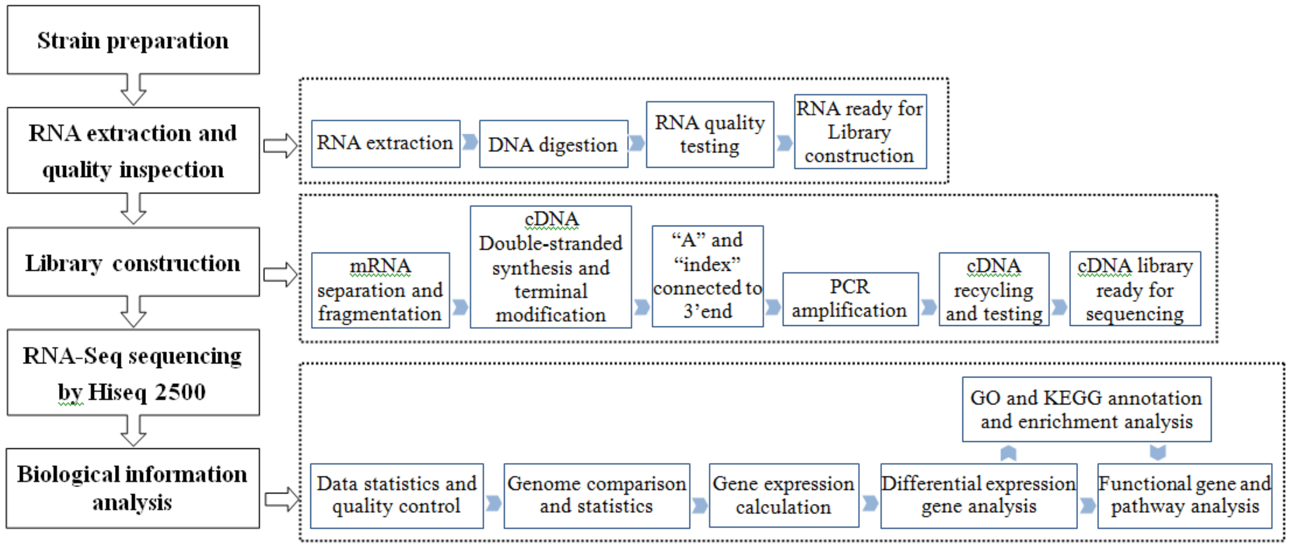

Fig. S1 Flow of RNA-Seq transcriptome sequencing and informatics analysis

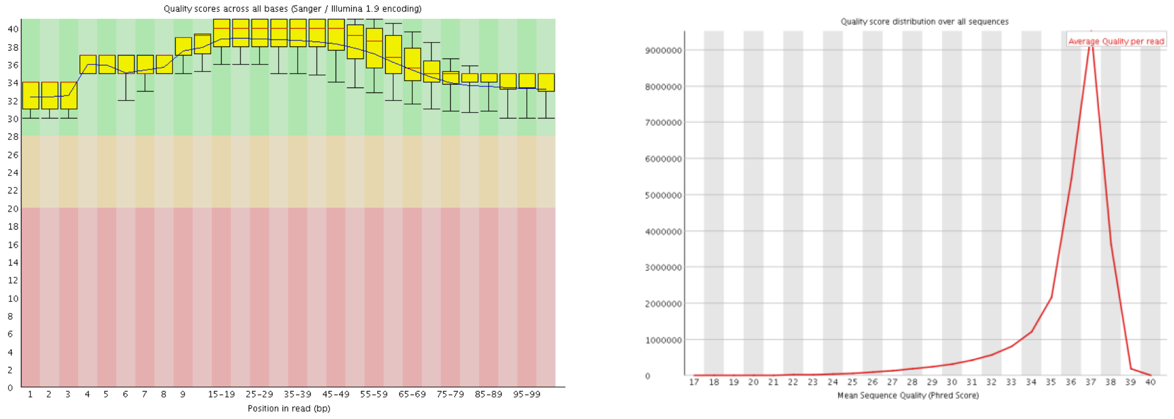

(a) H36

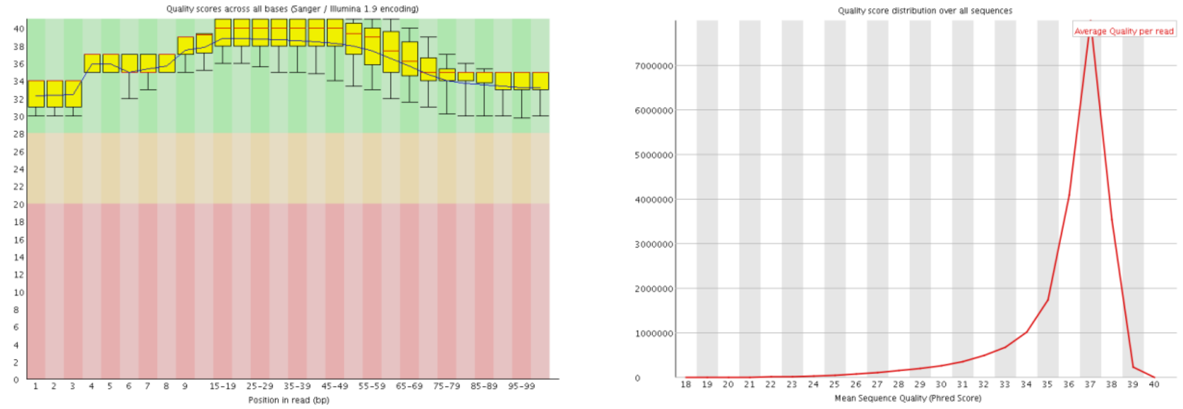

(b) L36

Fig. S2 Sequencing data quality control results under different nitrogen conditions

(a) sufficient nitrogen, C/N = 10:1 (b) nitrogen deficiency, C/N = 30:1

Table S1 Key enzymes in MBFA9 synthesis and properties

**Enzyme**	**Gene**	**Substrate**	**Product**	**Class**
2.7.1.2	glk	D-glucose	glucose 6-phosphate	Transferases
5.4.2.2	pgm	glucose 6-phosphate	glucose 1-phosphate	Isomerases
2.7.7.9	galU/galF	glucose 1-phosphate	UDP-glucose	Transferases
5.1.3.2	galE	UDP-glucose	UDP-galactose	Isomerases
1.1.1.22	ugd	UDP-glucose	UDP-glucuronate	Oxidoreductases
2.7.7.27	glgC	glucose 1-phosphate	ADP-glucose	Transferases
2.4.1.21	glgA	ADP-glucose	amylose	Transferases
2.4.1.18	glgB	amylose	glycogen	Transferases
3.2.1.1	amyA	glycogen	dextrin	Hydrolases
3.2.1.10	malL	dextrin	D-glucose	Hydrolases
5.3.1.9	pgi	glucose 6-phosphate	fructose 6-phosphate	Isomerases
2.6.1.16	glmS	fructose 6-phosphate	glucosamine 6-phosphate	Transferases
5.4.2.10	glmM	glucosamine 6-phosphate	glucosamine 1-phosphate	Isomerases
2.3.1.157	glmU	glucosamine 1-phosphate	N-acetyl-glucosamine 1-phosphate	Transferases
2.7.7.23	glmU	N-acetyl-glucosamine 1-phosphate	UDP-N-acetyl-glucosamine	Transferases
5.1.3.14	wecB	UDP-N-acetyl-glucosamine	UDP-N-acetyl-mannosamine	Isomerases
2.5.1.7	murA	UDP-N-acetyl-glucosamine	UDP-N-acetyl-glucosamine-enopyruvate	Transferases
1.3.1.98	murB	UDP-N-acetyl-glucosamine-enopyruvate	UDP-N-acetylmuramate	Oxidoreductases
5.3.1.8	manA	fructose 6-phosphate	mannose 6-phosphate	Isomerases
5.4.2.8	manB	mannose 6-phosphate	mannose 1-phosphate	Isomerases
2.7.7.13	manC	mannose 1-phosphate	GDP-mannose	Transferases
4.2.1.47	gmd	GDP-mannose	GDP-dehydro-rhamnose	Lyases
1.1.1.271	fcl	GDP-dehydro-rhamnose	GDP-beta-L-fucose	Oxidoreductases
2.4.1.83	DPM1	GDP-mannose	Dol-P-Man	Transferases
